# Are non-human primates capable of rhythmic entrainment? Evidence for the gradual audiomotor evolution hypothesis

**DOI:** 10.3389/fnins.2013.00274

**Published:** 2014-01-17

**Authors:** Hugo Merchant, Henkjan Honing

**Affiliations:** ^1^Department of Cognitive Neuroscience, Instituto de Neurobiología, Universidad Nacional Autónoma de México, Campus JuriquilaQuerétaro, México; ^2^Amsterdam Brain and Cognition, Institute for Logic, Language and Computation, University of AmsterdamAmsterdam, Netherlands

**Keywords:** rhythmic entrainment, macaques, interval timing, music origins

## Abstract

We propose a decomposition of the neurocognitive mechanisms that might underlie interval-based timing and rhythmic entrainment. Next to reviewing the concepts central to the definition of rhythmic entrainment, we discuss recent studies that suggest rhythmic entrainment to be specific to humans and a selected group of bird species, but, surprisingly, is not obvious in non-human primates. On the basis of these studies we propose the gradual audiomotor evolution hypothesis that suggests that humans fully share interval-based timing with other primates, but only partially share the ability of rhythmic entrainment (or beat-based timing). This hypothesis accommodates the fact that non-human primates (i.e., macaques) performance is comparable to humans in single interval tasks (such as interval reproduction, categorization, and interception), but show differences in multiple interval tasks (such as rhythmic entrainment, synchronization, and continuation). Furthermore, it is in line with the observation that macaques can, apparently, synchronize in the visual domain, but show less sensitivity in the auditory domain. And finally, while macaques are sensitive to interval-based timing and rhythmic grouping, the absence of a strong coupling between the auditory and motor system of non-human primates might be the reason why macaques cannot rhythmically entrain in the way humans do.

## Introduction

Rhythmic entrainment refers to the ability to perceive the pulse that marks equally spaced points in music or a sequence of auditory stimuli, and, then, to align the motor actions to that pulse or beat (Large and Palmer, 2002). Thus, the ability to perceive this pulse and synchronize to it (e.g., by foot tapping or dancing) is a common and widespread human skill (Wallin et al., 2000). It is also a skill that has been suggested to be species-specific (Fitch, 2009) and, arguably, specifically dependent on human developmental and/or evolutionary processes (Honing, 2012).

Rhythmic entrainment in humans involves timed movements of different body parts (such as finger, foot taps, or body sway) that occur rapidly and spontaneously, and that shows two important features: it matches the musical beat in both period and phase. These two aspects of rhythmic entrainment are conceptually distinct. Tempo/period matching means that the period of movement equals the musical beat period. Phase matching means that rhythmic movements occur near the onset times of musical beats. The intervals between each movement and the corresponding beat are called asynchronies and, in general, they acquire small negative values in humans indicating that synchronization is based on temporal anticipation (Repp, 2005). In fact, humans shows a remarkable ability to adjust, in both period and phase, their rhythmic action for a wide range of tempi and to complex musical signals with a changing tempo (Large and Jones, 1999). Furthermore, human listeners can synchronize at rates which are integer multiples or fractions of the basic beat (Honing, 2013). This indicates that the human mind has access to several distinct levels of periodicity, one of which can be selected at any given time as the beat (Drake et al., 2000). Therefore, rhythmic entrainment in human subjects is a complex cognitive phenomenon that depends in a dynamic interaction between the auditory and the motor systems in the brain (Grahn and Brett, 2007). It is important to mention that, although several insect and frog species synchronize their sound production with conspecifics during rhythmic chorusing, these species do not show some key features of rhythmic entrainment, such as the large flexibility to synchronize to a wide range of tempi (Large, 2000; Fitch, 2009).

## Is rhythmic entrainment species-specific?

For long, humans have been considered the only species capable of spontaneous synchronization of body movements with an auditory rhythmic pulse. However, recent studies have revealed that given a complex musical stimulus, animals that show vocal learning seem to be able to extract the beat and entrain their movements to it (Patel et al., 2009; Schachner et al., 2009; Hasegawa et al., 2011). Since both vocal learning and rhythmic entrainment depend on the tight coupling between the auditory and the motor systems to perceive and produce the desired movements, it has been hypothesized that the human capacity for rhythmic entrainment could be a by-product of the vocal learning mechanisms that allow us to learn speech sounds and musical melodies (Patel, 2006; Patel et al., 2009). However, one of the key studies supporting this hypothesis, performed by Patel et al. (2009) on a sulphur-crested cockatoo (*Cacatua galerita*) called Snowball, showed non-random phase relationships with the beat of music for very short windows of time (on average 25% in the observed bouts, with a range of 10–51%). Hence, this parrot showed only occasional periods of synchronization in response to music. In addition, no evidence of rhythmic entrainment was found in many other vocal learners (including dolphins, bats, and songbirds; Schachner et al., 2009), suggesting that vocal learning may be necessary, but clearly is not sufficient to observe rhythmic entrainment. Note, however, that since the Schachner et al. study (2009) was based on videos available on YouTube, the lack of videos for some species may not be an accurate assessment of their rhythmic capacities. Finally, a recent study (Cook et al., 2013) challenges vocal learning as a pre-condition to rhythmic entrainment, showing entrainment in a sea lion (*Zalophus californianus*) not considered a vocal mimic (Peterson and Bartholomew, 1969). Nevertheless, a formal study demonstrating that sea lions are indeed non-vocal-learners is still missing. What is clear in all these studies is their large differences in terms of: (1) the stimuli used to drive the behavior, (2) the tempi of the stimuli, (3) whether a constant or a variable beat is used, as well as (4) whether the animals have been trained on a synchronization task or they show spontaneous synchronization, and in the case of trained animals (5) how they have been trained and for how long. Hence, more experimental evidence is needed to reject or accept the vocal learning hypothesis. Furthermore, it is quite possible that the hypothesis that vocal learning is conditional on rhythmic entrainment, and hence predicting that it is absent in non-vocal learners, might be too bold of a distinction. As complex-vocal learning might have evolved in a more gradual fashion than thought before (Petkov and Jarvis, 2012), it might well be that the vocal learning hypothesis should be replaced by a hypothesis where the relation between vocal-learning and rhythmic entrainment depends on the gradual development of auditory-motor skills across evolution.

In the current paper we describe how Rhesus monkeys (*Macaca mulatta*) show some of the behavioral traits that define rhythmic entrainment but not all of them. Thus, we suggest that macaques cannot properly entrain their movements to an auditory beat because their motor system does not have the same access to auditory information as humans do.

## The gradual audiomotor evolution hypothesis

**Figure 1:**
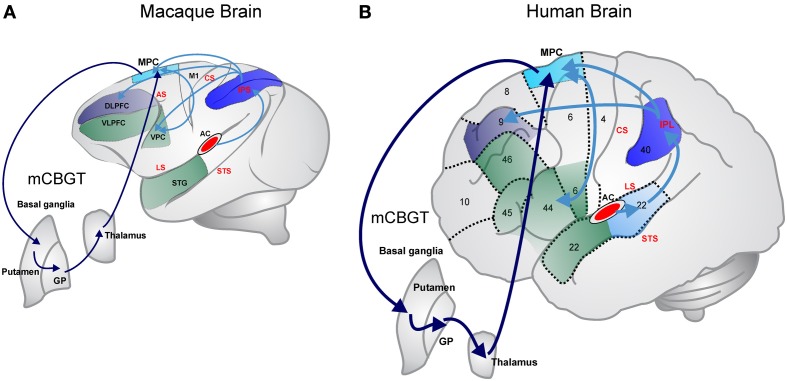
**Dorsal auditory stream in primates (marked in blue) that focus on processing sequential and temporal information. (A)** In the Rhesus monkey; **(B)** in the human. The dorsal stream starts in the interior/posterior parietal cortex that strongly connects with the medial and dorsal premotor areas, which in turn are reciprocally connected with ventral premotor and Broca's areas. Parietal areas also are connected with the dorsolateral prefrontal cortex. Medial premotor areas form the skeletomotor loop of the mCBGT circuit (cyan lines). The ventral stream associated with auditory object recognition is marked in green in both panels. AC, auditory cortex; DLPFC, dorsolateral prefrontal cortex; GP, globus pallidus; IPL, inferior parietal lobule; MPC, medial premotor cortex; VPC, ventral premotor cortex; VLPFC, ventrolateral prefrontal cortex; AS, arcuate sulcus; CS, central sulcus; IPS, inferior parietal sulcus; LS Lateral sulcus; STS, superior temporal sulcus; numbers in **(B)** correspond to Broadman's areas.

Functional imaging studies in humans have revealed that the motor cortico-basal ganglia-thalamo-cortical circuit (mCBGT; see dark blue arrow in Figure 1) is involved not only on sequential (Grafton et al., 1995) and temporal (Harrington et al., 2010; Wiener et al., 2010) processing, but also on rhythmic behaviors such as music and dance, where the auditory modality plays a critical role (Grahn and Brett, 2007). This circuit is usually involved in the control of voluntary skeletomotor movements and includes the supplementary motor cortex (SMA) and the putamen as the fundamental cortical and neostriatal nodes, respectively (Coull et al., 2011). Interestingly, neurophysiological studies in monkeys have also shown that the mCBGT circuit is engaged in both the perceptual and motor aspects of timing (Merchant et al., 2013a; Perez et al., 2013), as well as the control of movement sequences (Tanji, 2001).

The fact that macaques can accurately quantify single intervals and perform complex movement sequences, the key elements of rhythmic entrainment, made us believe that they could entrain their tapping movement to isochronous stimuli. However, the behavioral performance of Rhesus monkeys after a long period of training on a synchronization and continuation task (SCT) had mixed properties (Zarco et al., 2009). On one side, Rhesus monkeys show appropriate tempo matching, with movement periods that slightly underestimated the sensory metronome periods (~50 ms) for a range of intervals from 450 to 1000 ms. In addition, the variability of the intertap intervals increased linearly as a function of the interval, with slopes that are similar between macaques and human subjects. Hence, humans and monkeys share the ability of producing sequences of temporalized tapping movements. On the other side, macaques do not show phase matching, with tapping movements always lagging after the onset times of the metronome for ~250 ms. Nevertheless, the monkeys' asynchronies are smaller during the SCT than their reaction times to stimuli with a random interonset interval (600–1400 ms), indicating that these animals showed a predictive rhythmic behavior during the SCT (Zarco et al., 2009). Furthermore, in a recent study where the speed profile of the tapping movements was computed using semiautomatic video tracking algorithms, we have demonstrated that monkeys temporalize their movement-pauses and not their tapping movements during the SCT (Donnet et al., in press). Macaques showed a strong ability to temporalize their movement-pauses for a wide range of intervals (450–1000 ms), while their movements were similar across the duration of produced intervals, the sequential structure of the SCT, or the modality of the interval marker. These observations support the notion that monkeys used an explicit timing strategy to perform the SCT, where the timing mechanism controlled the duration of the movement-pauses, while also triggered the execution of stereotyped pushing movements across each produced interval in the rhythmic sequence (Donnet et al., in press).

Even though the observed asynchronies show that monkeys are not simply reacting to the sensory metronome, they do not show the negative mean asynchrony (NMA) that is commonly found in humans (Repp, 2005). This raises some important issues that need clarification. First, the reward contingencies during monkey training were focused on the tempo matching, so that they received a reward if each of the intervals produced had an error <35% of the target interval, and they could receive a double reward if the intertap interval was <20% of the target interval (Zarco et al., 2009; Merchant et al., 2011). We made a large effort to lower this high error threshold, but monkeys were not able to complete the 6 intervals in a trial with lower values, particularly during the continuation phase of the task, where monkeys were internally timing their tapping. Second, unpublished observations from our laboratory on two monkeys trained on a synchronization task with five produced intervals in sequence were designed to lower the tempo matching error for reward and added a threshold of phase-matching, so that asynchronies above a certain value were not rewarded. Using this simplified version of the task we found that the asynchronies could only reach values of ~100 ms after the stimulus on average, which confirms that macaques are not able to show the NMA observed in human subjects under the same circumstances. Third, since the range of intervals tested in SCT could not include the preferred tempo for rhythmic tapping in macaques, we tested a larger range of intervals in the simplified synchronization task. The results of these unpublished observations showed that Rhesus monkeys do not show tempo matching below 350 ms or above 1000 ms (see also Konoike et al., 2012). Finally, it is important to mention that NMA in humans depends on multiple factors, including stimulus complexity, the tempo of the auditory sequence, and whether the subjects have musical training or not (Repp, 2005; Honing, 2013). Consequently, the fact that macaques do not show NMA during different versions of the synchronization task does not demonstrate unequivocally that monkeys are incapable of rhythmic entrainment.

On the other hand, the training period for the complete SCT task in monkeys took more than a year to complete, and it was evident from the start that macaques had a large bias toward visual rather than auditory cues to drive their tapping behavior (Zarco et al., 2009). Again, unpublished observations on monkeys trained on the simplified version of the synchronization task, with tempo and phase-matching reward restrictions, showed long training periods to reach appropriate rhythmic behavior and a strong bias toward the visual modality. Consequently, the long training period for the complete execution of the SCT suggests that even if Rhesus monkeys can develop (through practice) strong prediction abilities (Zarco et al., 2009; Konoike et al., 2012), these animals are not naturally equipped to produce a sequence of temporalized movements. These observations also suggest that the neural circuit engaged in the control of this behavior cannot generate movements that coincide with the isochronous sensory metronome, and support the notion that monkeys have a preference for visuomotor rather than audiomotor integration.

The absence of synchronized movements to sound (or music) in certain species is no evidence for the absence of beat perception. With behavioral methods that rely on overt motoric responses it is difficult to separate between the contribution of perception and action; more direct, electrophysiological measures such as event-related brain potentials (ERPs), allow testing for neural correlates of beat perception (a pre-condition to rhythmic entrainment). To test this, we measured auditory ERPs in rhesus monkeys using the mismatch negativity component (MMN) as an index of (the violation of) rhythmic expectation (Honing et al., 2012). Rhythmic expectation was probed by selectively omitting parts of a musical rhythm, randomly inserting gaps at the first position of a musical unit (i.e., the “downbeat”). This oddball paradigm was used previously to probe beat perception in human adults and newborns (Honing et al., 2009; Winkler et al., 2009). The results confirmed the behavioral studies discussed earlier, in that Rhesus monkeys are not able to detect the beat in a complex auditory stimulus, although they can detect the start of a rhythmic group (Honing et al., 2012). In fact, a recent paper showed that macaques exhibit changes of gaze and facial expressions when a deviant of a regular rhythmic sequence is presented, supporting the notion that monkeys are sensitive to simple rhythms (Selezneva et al., 2013).

The question remains of whether closer human relatives such as the great apes, show a more sophisticated ability for rhythmic entrainment than macaques. While the vocal learning hypothesis predicts that no rhythmic entrainment should be found, a recent study (Hattori et al., 2013) showed that at least one chimpanzee (*Pan troglodytes*), of the three that took part in the experiment, was capable of spontaneously synchronizing her movements with an auditory rhythm. Interestingly, this chimpanzee entrained its tapping behavior to an isochronous 600 ms interval stimuli metronome, but not to other tempos.

Based on these observations we propose the *gradual audiomotor evolution hypothesis.* This hypothesis suggests rhythmic entrainment (or beat-based timing) to be gradually developed in primates, peaking in humans but present only with limited properties in other non-human primates; while humans share interval-based timing with all non-human primates and related species. Thus, this hypothesis accommodates the fact that the performance of rhesus monkeys is comparable to humans in single interval tasks (such as interval reproduction, categorization, and interception), but differs substantively in multiple interval tasks (such as rhythmic entrainment, synchronization, and continuation).

A recent study has shown that Japanese macaques (*Macaca fuscata*) are able to spontaneously synchronize their arm movements when they are paired and facing each other, suggesting that monkeys can coordinate their actions in a social setting and establish some level of rhythmic entrainment (Nagasaka et al., 2013). The asynchronies between the pairs of tapping monkeys are again positive, largely dependent on the visual input that the other monkey provides, and with little influence on the sounds that the monkeys made when tapping (Nagasaka et al., 2013). These observations support the notions that: (1) the visual modality plays a critical role in driving the rhythmic behavior of macaques, in accordance to the *gradual audiomotor evolution hypothesis*, and (2) behavioral imitation is an important source for social coordination.

## Single interval timing and visuospatial behavior are similar across primates

The interaction between sequential and temporal mechanisms in the auditory domain defines the ability for rhythmic entrainment. Single interval timing, in contrast, does not have a sequential component and can be carried out using auditory, visual, or somatosensory cues. Remarkably, macaques show equivalent temporal performance to humans in tasks that involve the perception and production of single intervals. For example, the relative psychometric threshold for the categorization of single intervals defined by brief visual cues is very similar between the two species (Mendez et al., 2011). Three different sets of intervals were used in blocks in that study, which allowed to determine whether humans and monkeys followed the scalar property of timing, a form of Weber law that states that the temporal variability increases linearly as a function of the interval (Gibbon et al., 1997). Indeed, the relative threshold increases linearly as a function of the interval, with similar slopes between the two primate species (Mendez et al., 2011). Also, the temporal performance between Rhesus monkeys and humans is similar during a single interval reproduction task, where subjects were trained to tap twice in a button in order to reproduce a previously trained interval defined by visual or auditory interval markers (Zarco et al., 2009). The intertap variances were comparable between species, with similar slopes in the linear increment of the intertap variance as a function of duration in the two primates (Zarco et al., 2009). It is important to note that the modality used to define the single intervals do not affect the temporal performance in both species (Zarco et al., 2009). Finally, humans and macaques show also similar abilities to intercept moving targets at different speeds (Merchant et al., 2003), computing the time remaining for the target to reach the interception zone in a similar fashion (Merchant et al., 2009). Therefore, the detailed psychometric comparisons between humans subjects and highly trained monkeys (using standard operant conditioning techniques) support the notion that the temporal abilities of both primates species during single interval tasks are rooted on a comparable neural system for the processing of single durations across different sensory-motor contexts (Merchant et al., 2013a).

The ability to use the spatial location of visual targets to execute sequential movements is also very similar between humans and macaques. Both species acquire sequential reaching procedures with similar learning curves and retain the motor skill for more than 1 year (Hikosaka et al., 2002). During the learning process of a particular sequence both primates changed from a reactive to a proactive mode, such that initially the hand and eyes moved after the target was illuminated and later the movements occurred before the target was tuned on (Hikosaka et al., 2002). Hence, macaques as well as humans have the capability to execute in a predictive fashion a long sequence of spatial reaching movements (up to 10 movements) toward visual cues. Indeed, the Rhesus monkey shows remarkable ability to deal with spatial information and its psychophysical similarity with human subjects at the perceptual (Britten et al., 1992; Romo et al., 2000), cognitive (Merchant et al., 2003, 2004; Fortes et al., 2004), and motor levels (Georgopoulos et al., 1986; Buneo et al., 2002). A number of combined neurophysiological and psychophysical experiments in macaques have been designed to uncover, with notable success, the functional organization of the neural circuits that mediates visuospatial processing (Hubel and Wiesel, 1968; Mountcastle et al., 1975; Georgopoulos et al., 1989). This neurophysiological information has been fundamental for understanding the human brain mechanisms of spatial behavior (Vanduffel et al., 2002; Kourtzi et al., 2003), because of the interspecies similarities in the visual system (Newsome and Stein-Aviles, 1999; Nichols and Newsome, 1999).

## Rhythmic entrainment in the auditory and visual domain

Speech, music, and dance include complex auditory stimuli to drive the perception or motor behavior in humans. Accordingly, during the SCT humans show smaller temporal variability and better accuracy with auditory rather than visual interval markers (Grondin et al., 1996; Repp and Penel, 2002; Merchant et al., 2008). In addition, when the senses deliver conflicting information, in humans vision dominates spatial processing, whereas audition dominates temporal processing (Repp and Penel, 2002; Bertelson and Aschersleben, 2003; Guttman et al., 2005). It has been suggested that the human perceptual system abstracts the rhythmic-temporal structure of visual stimuli into an auditory representation that is automatic and obligatory (Brodsky et al., 2003; Guttman et al., 2005). Thus, it is quite possible that the human auditory system has a privilege access to the temporal and sequential mechanisms working inside mCBGT circuit in order to determine the exquisite rhythmic abilities of the *Homo sapiens*.

In fact, the superior temporal areas of the cortex devoted to auditory processing in humans share massive reciprocal connections with premotor areas of the frontal lobe and project intensively to the neostriatum, the input of the basal ganglia (Yeterian and Pandya, 1998; Rilling et al., 2008; see Figure 1B). In contrast, the projections of these auditory temporal areas in the macaques to medial and ventral premotor areas (Petrides and Pandya, 1988; Rilling et al., 2008) and the basal ganglia are modest (Yeterian and Pandya, 1998; Borgmann and Jürgens, 1999; Thiebaut de Schotten et al., 2012; see Figure 1A). Furthermore, it has been suggested that the privileged access of the humans' auditory system to the sequential and temporal machinery of the mCBGT circuit emerged gradually in the course of evolution from precursors of the great ape lineage (Rauschecker and Scott, 2009). This notion is supported by a study showing that the connectivity between the superior temporal auditory areas and the frontal lobe in chimpanzees has an intermediate level of complexity when compared with macaques and humans (Rilling et al., 2008; Petrides and Pandya, 2009). It is clear, however, that the literature needs more systematic comparative studies on the connections between the auditory system with the basal ganglia and the frontal lobe (Rauschecker, 2012).

The diminished connectivity in the audio-premotor and audio-basal-ganglia circuits is accompanied by the well-known fact that motor behavior in macaques is more visual than auditory. For example, the accuracy of macaques for visual memory recognition does not extend to the auditory domain. Monkeys master the rule for one-trial recognition memory for visual stimuli extremely rapidly. Within several daily sessions, once they have learned the rule, monkeys show retention thresholds (performance at 75% accuracy) of 10–20 min after viewing a novel stimulus for only 1–2 s (Murray and Mishkin, 1984). In contrast, monkeys acquire the rule for one-trial memory exceedingly slowly for auditory stimuli, requiring a year or two of training before they can perform properly the task (Fritz et al., 2005). In macaques that can perform the auditory one-trial recognition memory task, the stimulus–retention thresholds are below 30–40 s after stimulus presentation (Fritz et al., 2005). Consequently, studies on movement sequences in monkeys always use visual-spatial cues to guide the behavior (Hikosaka et al., 1999; Tanji, 2001), and few experiments have reported the use of auditory stimuli as sensory signals to trigger arm motor responses (Tanji and Kurata, 1985; Matsuzaka and Tanji, 1996; Merchant et al., 2013a).

The similar timing performance for single intervals in all primates and the gradual increase rhythmic entrainment capabilities across anthropoids may depend on the neural systems that define the nested hierarchical properties of sequential and temporal behavior. Computational models have suggested three levels of hierarchical movements: the level of single motor acts or single sensorimotor associations; the level of simple action chunks, including either sequences of single motor acts or sensorimotor mappings; and finally, the level of superordinate action chunks composed of simple action chunks (Dehaene and Changeux, 1997). It appears that the mCBGT circuit in humans has different loops responsible for the concatenation of sequential auditory information or formation of “chunks,” (Graybiel, 2008; Leaver et al., 2009), and for temporal chunking of sensory information (Schubotz and von Cramon, 2001). Indeed, a functional imaging study has shown a gradient of activation that starts with the human anterior portion the Broca's area (area that and deeply involved in speech production) and its homolog in the right hemisphere during superordinate sequential chunks of learned finger movements, the posterior portion of these area for simple chunks, and the medial and dorsal premotor areas for single acts (Koechlin and Jubault, 2006). If we assume that superordinate sequential chunking is associated with rhythmic entrainment, the fact that monkeys show limited properties for rhythmic tapping may depend on their partial development of Broca's areas and their association with the basal ganglia and the premotor areas (Petrides and Pandya, 2009). This notion is supported by the observation that humans show a direct connection between the medial and ventral premotor areas and the posterior and anterior areas of Broca's, which is a smaller tract in macaques (Figure 1; Thiebaut de Schotten et al., 2012). Conversely, the similar abilities to perceive and execute single interval timing across primates may be due to the conserved functional-architecture of the medial and ventral premotor areas and the putamen that conform the classical skeletomotor mCBGT loop (Figure 1; Alexander et al., 1986). In this regard, recent studies have shown that the neurons of the primate SMA (Merchant et al., 2013b) and putamen (Bartolo et al., in press) are tuned to the intervals produced during the SCT. Furthermore, the preferred interval of a large population of cells was similar during the SCT and a single interval reproduction task, suggesting that these critical nodes of the mCBGT loop have an abstract representation of time during both behaviors (Merchant et al., 2013a). Hence, interval tuning can be an initial neural representation that could tag the duration of the produced intervals across the mCBGT circuit during single interval reproduction and the SCT, probably across the entire primate lineage.

Collectively, these empirical data suggest that the temporal processing within the monkey CBGT circuit show a preference for visual stimuli. Thus, we are explicitly suggesting that the lack of prevalence of the auditory system to engage the mCBGT circuit during behaviors that have a periodic and sequential structure results in a deficient rhythmic entrainment in macaques. It is clear, however, that macaques show some abilities to perform rhythmic behavior, and chimpanzees might be closer to the sophisticated rhythmic entrainment achieved by humans. By contrast, this circuit in monkeys is as capable as in humans to processes single intervals across different sensorimotor contexts and modalities.

## Vocal learning vs. gradual audiomotor evolution

The *gradual audiomotor evolution* and vocal learning hypotheses show the following crucial differences. First, the *gradual audiomotor evolution* hypothesis does not claim that the neural circuit that is engaged in rhythmic entrainment is deeply linked to vocal perception, production and learning, even if overlap between the circuits exists. Second, the *gradual audiomotor evolution hypothesis* suggests that rhythmic entrainment could have developed through a gradient of anatomofunctional changes on the interval-based mechanism to generate an additional beat-based mechanism, instead of claiming a categorical jump from non-rhythmic/single interval to rhythmic entrainment/multiple interval abilities. Third, since the CBGT circuit has been involved in beat-based mechanisms in imaging studies (Rao et al., 1997; Jäncke et al., 2000; Grahn and Brett, 2007; Wiener et al., 2010; Teki et al., 2011) we suggest that the reverberant flow of audiomotor information that loops across the anterior prefrontal CBGT circuits maybe the underpinning of human rhythmic entrainment. Finally, the *gradual audiomotor evolution* hypothesis suggests that the integration of sensorimotor information throughout the mCBGT circuit and other brain areas during the perception or execution of single intervals is similar in human and non-human primates. A crucial issue is that our hypothesis is considering that the perception and action of single intervals or rhythms are built-in the CBGT circuits as part of the same process (Merchant and de Lafuente, in press).

On the other hand, it is evident that all behaviors that have a complex periodic and sequential audiomotor structure, such as understanding other actions (mirror-neuron system), rhythmic entrainment, and speech, should engage partially overlapping networks that include the motor and prefrontal CBGT circuits, ventral premotor, and Broca's areas. In this regard, Mishkin and collaborators (Schulze et al., 2012) have suggested that auditory recognition of novel speech sounds occurs through the automatic transformation of the acoustic sequence into a subvocal oromotor sequence, and that this integrated acoustic/oromotor signal could then be stored as a lasting central representation. In concordance with the *gradual audiomotor evolution* hypothesis, these authors also suggest that the automatic transformation of the acoustic sequence into a subvocal oromotor sequence depends on the strong anatomical link between the dorsal auditory system and the frontal oromotor system (ventral premotor and Broca's areas), provided by the arcuate fasciculus in humans (Catani and Jones, 2005).

However, there are several important differences between the Gradual Audiomotor Evolution hypothesis and the motor theory of long term auditory memory proposed by Mishkin and collaborators (Schulze et al., 2012).

First, Schulze et al. (2012:7124) suggest that the highly proficient auditory long-term memory of humans is based on their ability to store rapidly fluctuating acoustic signals using the ventral premotor and the orofacial representation of the motor cortex, areas that are uniquely organized to chain-link rapid sequences and that are fundamental nodes of the speech and language circuits. We propose that the humans fully share interval-based timing with other primates, but only partially share the ability of rhythmic entrainment (or beat-based timing). Furthermore, it is in line with the observation that macaques can, apparently, synchronize in the visual domain, but show less sensitivity in the auditory domain.

Second, rhythmic entrainment explicitly engages the motor system to execute timed movements with high precision to the beat of an auditory sequence of stimuli. Schulze et al.'s theory implies that sounds used in memory tasks fluctuate at high-millisecond speeds and cannot be packaged for storage in the relevant sensory processing system alone, as in the case of static visual or tactile stimuli. Instead the recognition and storage of auditory stimuli needs the assistance of the oromotor system associated with language processing and execution.

Third, Schulze et al. suggest that an acoustic stimulus that can be neither mimicked nor labeled cannot be stored in long term memory for subsequent recognition (Schulze et al., 2012:7121). We discuss the need for more systematic empirical evidence to reject or accept the Vocal Learning hypothesis for rhythmic entrainment.

Fourth, Schulze et al. suggest that the recognition of the familiar words would require a form of episodic memory that depends on the interconnections of the superior temporal auditory processing stream, the lateral temporal semantic system, and the medial temporal lobe, including the hippocampus (Schulze et al., 2012:7122). We are not involving the ventral stream of auditory processing in the gradual audiomotor evolution hypothesis for rhythmic entrainment.

Finally, Schulze et al. hypothesize speech and auditory memory to be indissolubly linked that neither could have evolved without the other (Schulze et al., 2012:7124). We suggest that the gradual development of rhythmic entrainment is peaking in humans but is present only with limited properties in other non-human primates.

## Conclusion

We reviewed the literature on single and multiple interval timing tasks in humans and non-human primates, and observed different species to species behaviors between interval-based timing and beat-based timing, supporting the *gradual audiomotor evolution hypothesis*. This hypothesis accommodates the fact that the performance of Rhesus monkeys is comparable to humans in single interval tasks (such as interval reproduction, categorization, and interception), but differs in multiple interval tasks (such as rhythmic entrainment, synchronization, and continuation). The mCBGT circuit has a primary role in sequential and temporal processing, including rhythmic entrainment. However, it seems to be less engaged in audiomotor integration in Rhesus monkeys as opposed to humans (Grahn and Brett, 2007). While in a recent lesion study with humans (Grube et al., 2010) different cognitive mechanisms were shown to be active for interval-based timing vs. beat-based timing, with beat perception being dependent on distinct parts of the timing network in the brain (Merchant et al., 2011, 2013b; Teki et al., 2011), the anterior prefrontal CBGT and the mCBGT circuits in monkeys might be less viable to multiple interval structures, such as a regular beat. In contrast, macaques are as capable as humans to processes single intervals across different sensorimotor contexts and modalities.

### Conflict of interest statement

The authors declare that the research was conducted in the absence of any commercial or financial relationships that could be construed as a potential conflict of interest.
